# The Use of Cremation Data for Timely Mortality Surveillance During the COVID-19 Pandemic in Ontario, Canada: Validation Study

**DOI:** 10.2196/32426

**Published:** 2022-02-21

**Authors:** Gemma Postill, Regan Murray, Andrew S Wilton, Richard A Wells, Renee Sirbu, Mark J Daley, Laura Rosella

**Affiliations:** 1 Department of Epidemiology and Biostatistics Western University London, ON Canada; 2 Office of the Chief Coroner for Ontario Toronto, ON Canada; 3 Epidemiology Division Dalla Lana School of Public Health Toronto, ON Canada; 4 Public Health Agency of Canada Toronto, ON Canada; 5 Population and Public Health Research Program Institute for Clinical and Evaluative Sciences Toronto, ON Canada; 6 The Vector Institute for Artificial Intelligence Toronto, ON Canada; 7 Institute for Better Health Trillium Health Partners Mississauga, ON Canada

**Keywords:** excess deaths, real-time mortality, cremation, COVID-19, SARS-CoV-2, mortality, estimate, impact, public health, validation, pattern, trend, utility, Canada, mortality data, pandemic, death, cremation data, cause of death, vital statistics, excess mortality

## Abstract

**Background:**

Early estimates of excess mortality are crucial for understanding the impact of COVID-19. However, there is a lag of several months in the reporting of vital statistics mortality data for many jurisdictions, including across Canada. In Ontario, a Canadian province, certification by a coroner is required before cremation can occur, creating real-time mortality data that encompasses the majority of deaths within the province.

**Objective:**

This study aimed to validate the use of cremation data as a timely surveillance tool for all-cause mortality during a public health emergency in a jurisdiction with delays in vital statistics data. Specifically, this study aimed to validate this surveillance tool by determining the stability, timeliness, and robustness of its real-time estimation of all-cause mortality.

**Methods:**

Cremation records from January 2020 until April 2021 were compared to the historical records from 2017 to 2019, grouped according to week, age, sex, and whether COVID-19 was the cause of death. Cremation data were compared to Ontario’s provisional vital statistics mortality data released by Statistics Canada. The 2020 and 2021 records were then compared to previous years (2017-2019) to determine whether there was excess mortality within various age groups and whether deaths attributed to COVID-19 accounted for the entirety of the excess mortality.

**Results:**

Between 2017 and 2019, cremations were performed for 67.4% (95% CI 67.3%-67.5%) of deaths. The proportion of cremated deaths remained stable throughout 2020, even within age and sex categories. Cremation records are 99% complete within 3 weeks of the date of death, which precedes the compilation of vital statistics data by several months. Consequently, during the first wave (from April to June 2020), cremation records detected a 16.9% increase (95% CI 14.6%-19.3%) in all-cause mortality, a finding that was confirmed several months later with cremation data.

**Conclusions:**

The percentage of Ontarians cremated and the completion of cremation data several months before vital statistics did not change meaningfully during the COVID-19 pandemic period, establishing that the pandemic did not significantly alter cremation practices. Cremation data can be used to accurately estimate all-cause mortality in near real-time, particularly when real-time mortality estimates are needed to inform policy decisions for public health measures. The accuracy of this excess mortality estimation was confirmed by comparing it with official vital statistics data. These findings demonstrate the utility of cremation data as a complementary data source for timely mortality information during public health emergencies.

## Introduction

Quantifying the impact of COVID-19 on all-cause mortality in a timely manner is critical for understanding the full effect of the COVID-19 pandemic and for enabling evidence-based policy responses. While COVID-19 mortality in Ontario has been routinely tracked and reported through public health databases in near real-time, the reporting of Canadian vital statistics all-cause mortality data for Ontario, prior to the COVID-19 pandemic, was delayed by over a year [1]. Several challenges, including the need to centralize data, verify records, and categorize causes of death, impede using vital statistics data for real-time mortality surveillance [2]. Despite efforts to accelerate mortality reporting in the COVID-19 pandemic, significant data lags persist [3].

Cremation records, however, can be used to provide interim estimates of all-cause mortality [4,5]. This is because, before a cremation can occur, a coroner’s certification is required. As a result, cremation data are available in real-time, offering a consistent data source to examine mortality trends in a timelier manner. Early in the first wave (April to June 2020), cremation data detected an increase in all-cause mortality in Ontario, Canada [5]. These findings parallel the increases in mortality observed by several other countries during the first wave of the COVID-19 pandemic [6-10].

Several months later, Statistics Canada published mortality data that also demonstrated an increase in mortality in Ontario during the first wave (April to June 2020) [11]. Despite the detection of excess mortality, few studies systematically examine the performance of cremation data and specifically assess its utility as a real-time mortality surveillance tool. Lacking real-time mortality data posed a challenge for policy response since the magnitude of the mortality impact was unknown and thus influenced mitigation strategies that were implemented. With official mortality statistics available for the first wave of the COVID-19 pandemic, it is now possible to examine the extent to which cremation data accurately estimated the increase in all-cause mortality. This knowledge will be critical in informing whether cremation data can be leveraged as a timelier source of mortality information. Therefore, the objective of this study was to validate the use of cremation data as a surveillance tool for all-cause mortality during a public health emergency in a jurisdiction with delays in vital statistics data. Specifically, this study aimed to validate cremation records by determining (1) the stability of the percent cremated (ie, whether the percent cremated fluctuates by season and/or changes during the pandemic), (2) the timeliness of cremation records, and (3) the robustness/predictive ability of cremation records, measured by their ability to provide accurate estimations of all-cause mortality. The choice of such a measure aligns with the Centers for Disease Control and Prevention’s guidelines for evaluating surveillance tools [12]. The framework includes more metrics than the 3 operationalized here; however, this paper focuses on the aforementioned 3 components, given that they were previously unknown and are key to validating the use of cremation data as a surveillance tool.

## Methods

In Ontario—Canada’s most populous province—a cremation certificate must be provided by a coroner to authorize the cremation of a deceased person [13]. A licensed crematorium operator cannot proceed without a cremation certificate. The law in Ontario requires that a coroner review the circumstances surrounding the death before cremation takes place [2,13]. The certificate’s review and authorization are documented and kept on file at the crematorium [13]. Since 2017, these records have been collected and stored electronically by the Office of the Chief Coroner for Ontario. This database contains names, dates of birth and death, location of death, and cause of death for every person cremated in the province.

The Canadian Vital Statistics Death database, coordinated by Statistics Canada, collects demographic and cause of death information from all Canadian provinces and territories [14]. The cause of death is classified using the underlying cause of death according to the International Classification of Diseases 10th revision (ICD-10) [14]. The deaths captured in the data set comprise Canadian residents and nonresidents whose deaths occurred in Canada [14]. Routine data processes, including the submission and centralization of death certificates, verification of data, and coding of the cause of death, result in lags in the publication of mortality information [14].

All 323,988 cremations that occurred between January 1, 2017, and May 25, 2021 (with dates of death before April 30, 2021) were deidentified and maintained in an electronic database for the purpose of the analysis. The following analysis was done using Python 3.8.0. A small number of records (n=74) had the cause of death specified as “test” or age at death greater than 120 years, as they were false data used to set up the data set in 2017; these were identified and excluded from the analysis. The records were categorized according to the month of death and subcategorized by age and sex. Age is recorded as a numerical field, but it was converted into a categorical vector (0-44 years, 45-64 years, 65-84 years, and 85+ years) for the purpose of this analysis.

First, the utility of cremation data for surveillance was assessed by determining the percent cremated for the entire population and then by age and sex (Multimedia Appendix 1). In order to identify any seasonality in the proportion of death cremation, the percentage of deaths cremated was calculated for each week and for the annual quarters over the time period of 2017 to 2021. Standardized differences were used to assess the effect size of any variability in the percent cremated, given that they are independent of sample size [15]. The standardized difference is the difference in the mean of a variable between 2 groups divided by an estimate of the standard deviation of that variable [15]; relatively low values of the standardized difference show stability in the percent cremated.

Second, time lags in the completeness of cremation and vital statistics data were assessed to determine the predictive value of cremation data in estimating all-cause mortality. The data completion time lags were defined and calculated as the number of weeks between the date of death and when the data source contained >95% or >99% of the deaths that occurred in that week. Statistics Canada releases both provisional estimates of mortality and provisional counts of mortality; the latter was used in this study so that the analysis equivalents (ie, counts of cremation records to counts of vital statistics records) and findings could be generalizable to moments when there is uncertainty in a public health emergency’s effect on the reporting of vital statistics data. The data lag for Ontario’s vital statistics death data was assessed by comparing the provincial mortality reports released monthly by Statistics Canada [11] between July 24, 2020, and May 14, 2021, as seen in Multimedia Appendix 2. The percentage of the weekly mortality captured in each release was calculated using the weekly totals in the May 14, 2021, release as the denominator. The average time lags for both 95% and 99% completeness were calculated. The same method was applied to the weekly totals of the weekly data cuts of cremation data between May 1, 2020, and October 23, 2020, which were compared to the total number of deaths during that time as available in May 2021.

Third, to assess the predictive ability of cremation records, deviations from provincial mortality trends were quantified and compared both quantitatively and qualitatively to the available vital statistics data [3]. Excess mortality was defined and calculated as the population standardized percentage increase in the number of cremations, which was calculated using risk ratios (RRs) (percent increase = RR – 1), where the risk was cremation within the population of Ontario. Cremation records during 2020 and the first half of 2021 were compared to historical records from 2017 to 2019, grouped according to the month of death, and the age and sex of the decedent.

Cremation rates, analogous to mortality rates, were calculated using Statistics Canada’s quarterly population estimates [3,16]. Both absolute differences and relative differences (estimated using rate ratios), as compared to historical data, were calculated. The quarterly incident rate ratio was calculated to determine whether the cremation rate changed significantly during the COVID-19 pandemic relative to previous years [3,16,17]. Following the release of vital statistics data, the same methodology of calculating excess mortality in cremation data was applied to the data.

The weekly number of cremations and vital statistics deaths [3] were plotted on side-by-side graphs with 2020-2021 data and baseline data (2017-2019). While the trends were initially congruent, exponential smoothing was used to reduce some of the noise (weekly variability) so that the trends were more comparable; exponential smoothing was specifically chosen as the method of smoothing given that the data were demonstrated to be nonstationary with the Augmented Dickey-Fuller test [18]. The Statsmodel Holt package was used to exponentially smooth the trends of all graphs (and all subsequent graphs created in the analysis), and the default additive model was changed to an exponential model with a fixed smoothing slope (=.2) and smoothing level (=.6) [18].

To assess whether confirmed COVID-19 deaths accounted for the entirety of the increase in deaths, excess mortality was calculated, for which COVID-19–classified deaths were removed from the cremation and vital statistics records. Deaths due to COVID-19 were isolated from the records by the presence of the terms “COVID,” “novel coronavirus,” “Sars-CoV-2,” and “coved-19” (a spelling typo) in the *cause of death*, *antecedent cause*, and *other cause* *of death* categories of the cremation records. Records that matched the above criteria but also contained the phrases “test results pending,” “possible,” “not,” “non,” or “negative” were excluded from the classification of death due to COVID-19.

A database containing cremation certificates is held at the Office of Chief Coroner for Ontario. The data-sharing agreement for this study prohibits the data from being publicly available. Data requests may be granted provided there is an appropriate data-sharing agreement in place.

### Ethics Approval

This study was approved by the Research Ethics Board of Western University (project ID: 112478).

## Results

**Figure 1:**
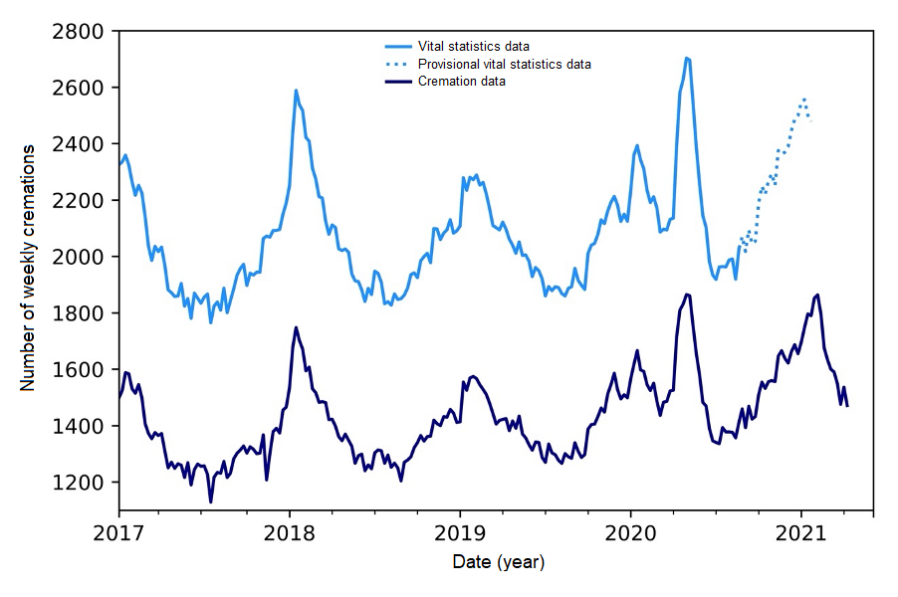
The weekly number of deaths in Ontario, Canada, as reported in Ontario’s cremation records (January 2017 to April 2021, considered >99% complete) and vital statistics records (January 2017 to December 2020, released May 2021). Given that vital statistics records from mid-August (August 16, 2020) and onwards are <95% complete, they are considered provisional. Their respective trends have been smoothed using the Statsmodel Holt package; the default additive model has been changed to an exponential model with a fixed smoothing slope (β=.2) and smoothing level (α=.6).

**Figure 2:**
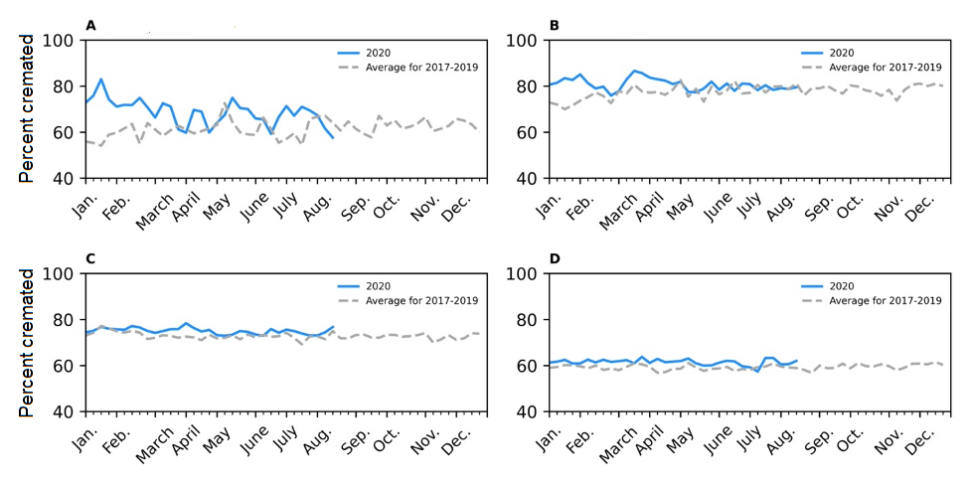
The weekly percent cremated in Ontario, Canada by age group for the average of 2017, 2018, and 2019, in comparison to the percent cremated in 2020. The age groups are as follows: 0-44 years (A), 45-64 years (B), 65-84 years (C), and 85 years or over (D). The respective trends have been smoothed using the Statsmodel Holt package; the default additive model has been changed to an exponential model with a fixed smoothing slope (β=.2) and smoothing level (α=.6). As a single year is compared to the average of 3 years, it was expected that 2020 will display a greater level of weekly fluctuation.

Since the cremation records became electronic (in 2017) and prior to the COVID-19 pandemic, the majority of Ontarians were cremated upon death, with 67.4% (95% CI 67.3%-67.5%) of Ontarians being cremated following death (Figure 1). There is no seasonality in the percent cremated, with the percent cremated remaining stable throughout the year. Thus, Ontario’s cremation data can capture patterns in all-cause mortality between 2017 and 2019, as evidenced in Figure 1. The percent cremated does not differ by sex (Multimedia Appendix 1). However, there are slight differences by age. The percent cremated is the greatest for those aged 45-64 years and 65-84 years, with approximately 75%-80% and 70%-75%, respectively, being cremated (Figure 2). The percent cremated for those aged 0-44 years and 85 years or over is approximately 60%.

The percentage of the population cremated remained stable during the COVID-19 pandemic, as seen in Table 1 and Figure 2. Stability was evident in the standardized difference calculations that compared the COVID-19 period to the historical period and obtained a value less than 10% difference. Stability in the percent cremated was also observed within each age group studied (0-44 years, 45-64 years, 65-84 years, and 85 years or over), as seen in Figure 2 and Multimedia Appendix 1.

**Table 1:** Stability in the percentage of Ontarians cremated, 2017-2020.

Variable				January to March^a^		April to June^a^		July to September^a^		October to December^a^		January to December
**Number of deaths, n**												
	**Baseline (2017-2019)^b^**											
		Cremation records	19,045		17,146		16,884		18,568		71,644	
		Vital statistics records^c^	28,540		25,443		24,947		27,405		106,335	
	**2020**											
		Cremation records	20,032		20,737		18,776		21,209		80,754	
		Vital statistics records^c^	28,675		29,750		N/A^d^		N/A		N/A	
**Percent cremated (%), value (95% CI)^e^**												
	2017-2019		66.7 (66.4-67.0)		67.4 (67.1-67.7)		67.7 (67.4-68.0)		67.8 (67.5-68.1)		67.4 (67.3-67.5)	
	2020		69.9 (69.6-70.2)		69.7 (69.4-70.0)		N/A		N/A		N/A	
	Standardized differences		6.88%		4.96%		N/A		N/A		N/A	

^a^For 2020, January to March was the prepandemic period, April to June was the first wave of the pandemic, July to September was summer, and October to December was the second wave of the pandemic.

^b^The average number of deaths in 2017, 2018, and 2019 during the same time period.

^c^The number of deaths in Ontario as reported by Statistics Canada in May 2021; at this time, Statistics Canada considers these numbers complete up to the end of July 2020 [3].

^d^N/A: not applicable.

^e^The 95% CI is calculated using the standard error for population proportions.

In addition, cremation data also provide a timelier source of mortality information, given that cremation records are available much sooner than vital statistics mortality records (Multimedia Appendix 2). On average, cremation records are >95% complete within 1 week of the date of death and >99% complete within 3 weeks. In contrast, the vital statistics data had an average delay of 27 weeks (range 23-31 weeks) for reporting 95% completeness and a 39-week delay for 99% completeness (range 34-43) (Multimedia Appendix 2). Thus, the vital statistics data published by Statistics Canada for August 2020 and onwards is <95% complete and, as Statistics Canada states, is provisional [11].

The real-time mortality estimates with cremation data showed, as early as June 2020, that there was excess all-cause mortality during the first wave of the COVID-19 pandemic (April to June 2020; Table 2). Specifically, when standardized for the provincial population, there was an increase of 16.9% (95% CI 14.6%-19.3%; n=+3591) during April to June when compared with the number of cremations from the previous years (2017-2019), as seen in Table 2. This is in contrast to the 1.7% increase (95% CI −0.3% to 3.7%; n=+987) seen in January to March of 2020 (Table 2). Using the provisional vital statics data released by Statistics Canada [11] and the same methodology for calculating excess mortality that was used for cremation data, there was a 13.1% increase (95% CI 11.2%-15.0%; n=+4307) in mortality during the first wave (April to June 2020).

**Figure 3:**
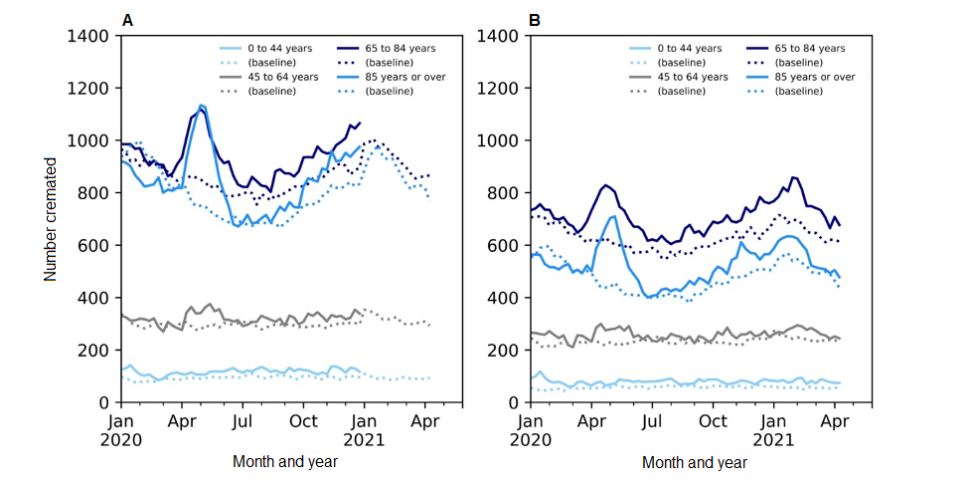
Side-by-side comparison of the weekly number of deaths in Ontario, Canada during the pandemic by age group as reported in (A) vital statistics data, which contains all provincial deaths and is released by Statistics Canada [3], and (B) Ontario’s cremation data for January 2020 to April 2021, which refers to the baseline data (the average of data in 2017-2019). The annual trends have been smoothed using the Statsmodel Holt package; the default additive model has been changed to an exponential model with a fixed smoothing slope (β=.2) and smoothing level (α=.6).

Cremation data even captured the trends in excess mortality at an age-specific level. When broken down by age, excess mortality during the first wave (April to June 2020) was observed among all age groups in both the cremation and vital statistics data (Figure 3; Multimedia Appendix 1). Cremation data accurately captured the magnitude of excess in each age group. In both data sets, the largest absolute increase occurred in the older age groups. In fact, 80.3% (1734/2373) of the excess deaths in the cremation records and 83.4% (3587/4302) of the vital statistics deaths during this time period were observed among individuals aged 65 years or older. It is also important to note that the greatest relative change in both cremation and vital statistics records occurred in those less than 45 years of age (Figure 3; Multimedia Appendix 1).

**Table 2:** Magnitude of excess mortality in Ontario, Canada identified with Ontario’s cremation records during the COVID-19 pandemic, January 2020 to March 2021.

Variable		January to March^a^	April to June^a^	July to September^a^	October to December^a^	January to December
**Baseline (2017-2019)^b^**						
	Number of cremations	19,045	17,146	16,884	18,568	71,644
	Rate of cremations per 100,000, value (95% CI)^c^	134 (132 to 136)	120 (119 to 122)	118 (116 to 120)	129 (127 to 131)	501 (497 to 504)
**2020**						
	Number of cremations	20,032	20,737	18,776	21,209	80,754
	Absolute change in the number of cremations^d^	987	3591	1892	2641	9110
	Population standardized percentage increase (%)^e^, value (95% CI)^f^	1.7 (−0.3 to 3.7)	16.9 (14.6 to 19.3)	8.0 (5.8 to 10.3)	11.6 (9.4 to 13.8)	12.7 (8.4 to 10.6)
	Rate of cremations per 100,000, value (95% CI)^c^	136 (134 to 138)	140 (139 to 143)	127 (126 to 129)	144 (142 to 146)	548 (544 to 552)
	Incident rate ratio^g^, value (95% CI)	1.02 (1.00 to 1.04)	1.17 (1.15 to 1.19)	1.08 (1.06 to 1.10)	1.12 (1.09 to 1.14)	1.09 (1.08 to 1.11)
**2021**						
	Number of cremations	21,418	N/A^h^	N/A	N/A	N/A
	Absolute change in the number of cremations^d^	2373	N/A	N/A	N/A	N/A
	Population standardized percentage increase (%)^e^, value (95% CI)^f^	8.2 (6.1 to 10.3)	N/A	N/A	N/A	N/A
	Rate of cremations per 100,000, value (95% CI)^c^	145 (143 to 147)	N/A	N/A	N/A	N/A
	Incident rate ratio^g^, value (95% CI)	1.08 (1.06 to 1.10)	N/A	N/A	N/A	N/A

^a^For 2020, January to March was the prepandemic period, April to June was the first wave of the pandemic, July to September was summer, and October to December was the second wave of the pandemic. For 2021, January to March and April to June involved the third wave.

^b^The average of the number of deaths in 2017, 2018, and 2019 during the same time period.

^c^Cremation rates, analogous to mortality rates, were calculated as the number of cremations divided by the provincial quarterly population estimates published by Statistics Canada [16].

^d^Absolute change refers to the difference in the number between 2020/2021 and the baseline (2017-2019).

^e^The population standardized percentage increase is calculated as risk ratio (RR) − 1, where RR is the incidence of death (measured as the number of cremation) in the quarterly population estimates. The Q3 population estimate was used for the January-December RR.

^f^The 95% CI for the percentage increase is calculated as (RR lower bound − 1) × 100% to (RR upper bound + 1) × 100%. The RR CI is calculated as =EXP(LN(RR) − (1.96 × SE)), with SE(lm(rr)) = sqrt(1/Ncrem_(2017-19)_ − 1/Npop_(2017-19)_ + 1/Ncrem_(2020)_ − 1/Npop_(2020)_).

^g^The quarterly incident rate ratio was calculated by dividing the rate of cremations in 2020 to that of the baseline. The 95% CI was calculated as =(Events ± 1.96 × SE) / population × 100,000, where SE is the standard error equal to the square root of the number of cremations [17].

^h^N/A: not applicable.

Most recently, in the latter half of 2020 (the second wave) and the beginning of 2021 (the third wave), there was still significant excess mortality (Table 2). Specifically, after adjusting for population, there was an 8.0% increase (95% CI 5.8%-10.3%) in mortality in the summer months (July to September 2020), a 11.6% increase (95% CI 9.4%-13.8%) in the fall (October to December 2020), and a 9.5% increase (95% CI 8.4%-10.6%) during January to March 2021, relative to the same period for previous years. During this time, the absolute unadjusted excess mortality among those aged 0-44 years accounted for a greater percentage of the overall excess mortality (22%-27% of the excess) than in the first wave (20% of the absolute unadjusted excess). In fact, unlike any other age group, excess mortality for those aged 0-44 years in the COVID-19 pandemic was the greatest in early 2021 (January to March). In contrast, during this time (January to March 2021), excess mortality for those aged 85 years or older was lower than at any other time in the COVID-19 pandemic (Table 2).

**Figure 4:**
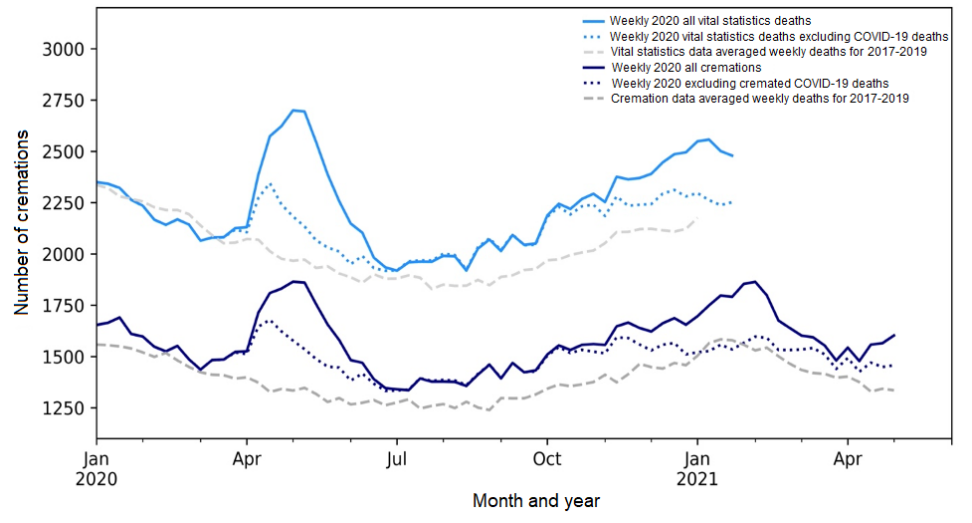
Annual trends of the weekly number of deaths with and without confirmed COVID-19 deaths for the cremation records and vital statistics data for Ontario, Canada (released May 2021). The pandemic waves in Ontario, Canada captured in this graph are as follows: wave 1 (April 2020 to June 2020), wave 2 (September 2020 to February 2021), and wave 3 (April 2021 onwards). Both trends have been smoothed using an exponential model with a fixed smoothing slope (β=.2) and smoothing level (α=.6).

Beyond simply highlighting excess mortality, cremation data demonstrated that, as early as June 2020, deaths due to COVID-19 could not explain all of the excess mortality observed during the pandemic (Figure 4; Multimedia Appendix 3). With vital statistics data available for the first wave, it is clear that cremation data accurately estimated the magnitude of excess non–COVID-19 mortality (Figure 4).

During the period from March 23, 2020, to July 5, 2020, cremation data captured 55.6% (1638/2945) of the provincially reported COVID-19 deaths, which is consistent with the percent cremated among those aged 85 years or older (the age group with the majority of COVID-19 deaths during this time). When these COVID-19 deaths were removed, there was still significant excess mortality during the last 3 quarters of 2020. Specifically, there was a 7.9% (95% CI 5.7%-10.1%) increase in April to June 2020, 7.5% (95% CI 5.3%-7.8%) increase in July to August 2020, and 5.9% (95% CI 3.9%-8.1%) increase in September to December 2020. Elevated non–COVID-19 mortality was not detected in the first quarter of 2021 (Multimedia Appendix 3).

## Discussion

Real-time mortality information is an essential indicator to monitor during the COVID-19 pandemic. Many jurisdictions experience delays in official vital statistics data due to verification processes, and as a result, it can be difficult to obtain timely mortality information. This study aimed to provide evidence of the utility of cremation data as an early indicator of mortality trends during the COVID-19 pandemic. Within 3 weeks, cremation data captured approximately 70% of overall mortality, anticipating vital statistics mortality records by approximately 5 months. Timeliness was of critical value given that during this time, several public health measures and policies related to the pandemic response were being decided in the absence of mortality data in many jurisdictions, including in Canada, where official vital statistics reports lag by several months.

Additionally, this analysis demonstrated that the percentage of the population cremated remained stable during the COVID-19 pandemic. This was a key finding given that, at the start of the pandemic, there was a concern as to whether a shift in burial practices drove the increase in cremations. This was, in fact, not the case and aligned with the fact that there was no change in the guidance of embalming. These findings provide further confidence for the use of cremation data to obtain a robust and real-time estimate of mortality during public health emergencies in jurisdictions where the death investigation system is equipped with digital reporting tools.

In demonstrating the ability of cremation data to capture population-level mortality trends, the analysis captured excess mortality, that is, mortality beyond what is normally anticipated relative to the average of the 3 prior years. COVID-19 cremations could not account for all of this excess. Excess mortality due to COVID-19 has been demonstrated in several countries, including the United States [19-21], Portugal [9], Sweden [22], England [23], and many others [6-8,10,24,25]. While overall excess mortality (identified by this study) during the first wave in Canada (April to June 2020) is lower than in some countries, such as Spain [8] and England [23], it is substantially higher than in other industrialized countries, including Germany [26], Sweden [22], and Norway. The age structure of excess mortality that we report here is similar to that found by the European monitoring of excess mortality for public health action (EuroMOMO) network, with the majority of the excess occurring in older age groups [7]. Other provinces in Canada, such as Alberta and British Colombia, also detected a similar magnitude of excess mortality in 2020 [11,27]. Consequently, cremation data can provide an understanding of the most recent mortality trends (the fourth and fifth waves), for which vital statistics data are still provisional.

In addition, our results confirming that COVID-19 deaths did not account for the entirety of the excess mortality in Ontario are supported by similar findings in several other countries [8,9]. Excess mortality that cannot be accounted for by confirmed COVID-19 is attributable to several factors, and these factors likely changed throughout the pandemic, reflecting the various patterns seen in Figure 4. The initial large increase in non–SARS-CoV-2 mortality during March to April 2020 was likely due to underdiagnosis and underreporting of COVID-19 on death certificates, delays in care for conditions other than COVID-19, including cancer and cardiovascular care, worsening mental health and substance use, and hesitancy or fear that deterred patients from seeking emergency care for cardiac events, all of which have been observed as causes of non–COVID-19 excess in other jurisdictions [28-35]. The decline in emergency care-seeking is supported by data showing that Canadian emergency department volumes dropped during the first wave of the COVID-19 pandemic [17,36]. Subsequent excess mortality, when COVID-19 testing was more widely employed, is likely due to the indirect effects of the pandemic, including acute drug toxicity, violence, and the economic and social disruptions of the pandemic [30,32,37]. Specifically, in Ontario, opioid-related deaths have increased by 79%, with the majority of these deaths occurring among those aged 25-44 years [37]. These increases in all-cause mortality highlight the value and necessity of real-time surveillance of population mortality rates, especially during public health emergencies. Given their robust and real-time nature, cremation data offer a critical source of mortality data that can provide these insights in the interim period before vital statistics data are available.

An important limitation of the study is the assumption of minimal growth in the population cremated. The quantifications of excess should be interpreted with these limitations in mind, particularly given that they contribute to the discrepancies in the reported values of excess mortality following the release of provisional vital statistics mortality data by Statistics Canada after several months, using the Farrington method. However, small discrepancies in the magnitude of excess mortality reported do not invalidate the use of cremation data, given that the value of a surveillance tool is in its ability to be both timely and accurate, and cremation data display strong congruency to vital statistics data, as seen in Figure 4. Likewise, with other data sources that can be used to create robust modes, the long reporting delays introduce significant biases. Thus, the methodology that is employed and able to communicate these results in a timely manner clearly shows the value in using Ontario’s cremation records to provide the earliest indication of excess all-cause mortality in a region with delays in vital statistics reporting.

There are some additional limitations to this analysis that should be considered. First, there was no advice against embalming during the COVID-19 pandemic; thus, in a public health emergency wherein embalming may be advised against, the utility of cremation data would need to be further investigated. Second, this study looked at all-cause mortality, and therefore, it is not possible to conclude whether COVID-19 affected the likelihood of being cremated. However, we found no evidence of this, given that there was no relationship between the cause of death and the likelihood of cremation. The next steps of this research include determining the methodology for using cremation data to assess the effect of the COVID-19 pandemic on other specific causes (ie, cardiac events) and manners (ie, suicide) of death. Finally, it is important to note that while cremation data represent most deaths in Ontario, they do not represent all deaths. Certain segments of the population may have an intrinsically higher or lower cremation rate (ie, people of Jewish faith). This is an important consideration, and if some segments of the population have different rates of cremation and different rates of mortality, the estimates would be biased. However, this is not an invalidating limitation for a surveillance model. Likewise, owing to the structure of the cremation data set (ie, absence of ethnic data), we were unable to assess whether the magnitude of excess mortality differed among different subgroups of the population. Despite these limitations, the results compellingly demonstrate the utility of cremation data as an important surveillance data source when timelier estimates of mortality are needed, such as during a public health emergency. The purpose of this analysis was to demonstrate cremation data as a robust estimator of all-cause mortality in public health emergencies in the interim of vital statistics reporting, and not as a replacement of vital statistics data or as a predictor of a pandemic’s effects on subpopulation mortality.

In conclusion, the COVID-19 pandemic emphasized the importance and need for real-time mortality information. Cremation data can be used for monitoring all-cause mortality in conjunction with vital statistics data. In addition, other jurisdictions with a lack of real-time mortality surveillance and high cremation rates will likely benefit from leveraging cremation data to estimate the complete impact of a public health emergency on all-cause mortality. This is an important finding, given that the utility of cremation data to estimate all-cause mortality in a public health emergency was previously unknown. This study demonstrates that cremation records can provide robust and timely indicators of all-cause mortality and should be used as interim mortality data during a public health emergency where more timely data can support the response.

## Appendix

Multimedia Appendix 1 Further analysis on the percent cremated by age and sex.

Multimedia Appendix 2 Maturation of both cremation data and vital statistics data.

Multimedia Appendix 3 Calculations for non–COVID-19 excess.

## Abbreviations

RR: risk ratio
