# Supplementary material for: The Use of Cremation Data for Timely Mortality Surveillance During the COVID-19 Pandemic in Ontario, Canada: Validation Study
Source: JMIR Public Health Surveill. 2022 Feb 21;8(2):e32426. doi: 10.2196/32426 (PMC8862761; doi:10.2196/32426)
Supplement: Multimedia Appendix 1 [file publichealth_v8i2e32426_app1.docx]

**Supplemental 1: Detailed Sex and Age Analysis**

The following supplemental details additional data to demonstrate the stability in the percent cremated and excess mortality by sex and age.

**Methodology**

Within the electronic cremation certificates, sex is a categorical variable. The options are male, female, or unspecified/unknown. In the 323,988 cremation records with dates of death between January 1, 2017 and April 30, 2021 (inclusively) 74 had missing or unknown values for the variable of sex; these were excluded from the age analysis.

Age, in contrast, is a numerical variable. Of the 323,988 cremation records with dates of death between January 1, 2017 and April 30, 2021 (inclusively) 6,885 (2%) had missing values for the variable of sex; these were excluded from the age analysis. Age was converted to a categorical variable for the analysis (0 to 44 years, 45 to 64 years, 65 to 84 years, and 85 years and older).

The same methodology used in the manuscript was applied here: records from the baseline year (2017-2019) were averaged, and the average was compared to 2020 and 2021. The same was done for Vital Statistics data and subsequently, the percent cremated was calculated. However, given that Vital Statistics Data is not available for the entirety of the pandemic, the percent cremated could only be calculated up to mid-2020, at which point Vital Statistics Data is considered complete.

**Results**

The results of the analysis demonstrate (1) stability in the percent cremated by sex and age category and (2) age-variability in excess mortality.

First, stability in the percent cremated. By sex, while the average precents of Ontarians cremated in the baseline (2017-2019) is slightly higher for men (69.4%) than that of women (65.2%) (Table 1A). This finding could be partly explained by the older age of death for females given our age analysis highlighted that the percent cremated for those over 85 years is lower than that of those 65 to 84 years. Regardless, the percent cremated remained consistent for both sexes throughout the pandemic; there was no significant change in the percent cremated during the first wave (Table 1A).

Likewise, by age, there was variability, as expected, in the percent cremated among each age group (Table 2A). Specifically, the percent cremated is greatest for those aged 45-64 years and 65-84 years, with approximately 75-80% and 70-75%, respectively, being cremated (Table 2A). The percent cremated for those 0-44 years as well as 85 years and over is approximately 60% (Table 2A). Consequently, cremation data is a representative source of population-level all-cause mortality information. This finding verifies that cremation data can be used to detect sex-specific and age-specific excess mortality during the COVID-19 Pandemic and subsequent pandemics that do not advice against embalming.

Second, while the percent cremated remained stable throughout the pandemic, the number cremated increased for both sexes. Overall, the unadjusted percent increase was slightly greater for men (+13.7%) than for women (+11.7%) (Table 1B). When broken down by age, excess mortality during the first wave (April to June 2020) was observed among all age groups in both the cremation and vital statistics data and cremation data accurately captured the magnitude excess in each age group (Table 2B). In both datasets, the largest absolute increase occurred in the older age groups. In fact, 80.3% of the excess deaths in the cremation records and 83.4% of the vital statistics deaths during this time period were observed among individuals aged 65 years and older (Table 2B). It is also important to note that the greatest relative change in both cremation and vital statistics records occurred in those less than 45 years (Table 2B).

1. **Sex Analysis Tables**

**Table 1A**. A demonstration of the stability in the percent of Ontarians cremated by sex, 2017-2020 and an analysis of the excess mortality detected in 2020-2021.

|  |  | **Timeframe (monthly)** | | | | |
| --- | --- | --- | --- | --- | --- | --- |
|  | **Sex** | **January to March** | **April to**  **June** | **July to September** | **October to December** | **January – December** |
| **Number of Deaths** | |  |  |  |  |  |
| ***Baseline (2017-2019)^1^*** | |  |  |  |  |  |
| Cremation Records | F | 9148 | 8081 | 8000 | 8787 | 34,016 |
|  | M | 9894 | 9060 | 8878 | 9777 | 37,610 |
| Vital Statistics Records^2^ | F | 14,165 | 12,392 | 12,158 | 13,457 | 52,172 |
|  | M | 14,375 | 13,052 | 12,788 | 13,948 | 54,163 |
| ***2020*** |  | ***Pre-Pandemic*** | ***First wave*** | ***Summer*** | ***Second wave*** |  |
| Cremation Records | F | 9571 | 9753 | 8719 | 9938 | 37,981 |
|  | M | 10,458 | 10,979 | 10,049 | 11,270 | 42,756 |
| Vital Statistics Records^2^ | F | 14,165 | 14,485 |  |  |  |
|  | M | 14,375 | 15,265 |  |  |  |
| ***2021*** |  | ***Third Wave*** |  |  |  |  |
| Cremation Records | F | 10,029 |  |  |  |  |
|  | M | 11,387 |  |  |  |  |
| **Percent Cremated** | | | | | | |
| Baseline  (2017-2019)^1^ | F | 64.6 | 65.2 | 65.8 | 60.7 | 65.2 |
|  | M | 68.8 | 69.4 | 69.4 | 70.1 | 69.4 |
| 2020 | F | 67.6 | 67.3 |  |  |  |
|  | M | 72.8 | 71.9 |  |  |  |

1 The average of the number and percent of deaths in 2017, 2018, and 2019 during the same time period.

2 The number of deaths in Ontario as reported by Statistics Canada in May 2021; these numbers are considered complete up to August 2020.^3^

**Table 1B**. A demonstration of the stability in the percent of Ontarians cremated by sex, 2017-2020 and an analysis of the excess mortality detected in 2020-2021.

|  |  | **Timeframe (monthly)** | | | | |
| --- | --- | --- | --- | --- | --- | --- |
|  | **Sex** | **January to March** | **April to**  **June** | **July to September** | **October to December** | **January – December** |
| **Absolute Increase^1,^** | | | | | |  |
| ***2020*** |  | ***Pre-Pandemic*** | ***First wave*** | ***Summer*** | ***Second wave*** |  |
| Cremation Records | F | 423 | 1672 | 719 | 1151 | 3965 |
|  | M | 564 | 1919 | 1171 | 1493 | 5146 |
| Vital Statistics Records^2^ | F | -40 | 2093 |  |  |  |
|  | M | 175 | 2213 |  |  |  |
| ***2021*** |  | ***Third Wave*** |  |  |  |  |
| Cremation Records | F | 881 |  |  |  |  |
|  | M | 1493 |  |  |  |  |
| **Percentage Increase^3^ (%)**  **(unadjusted)** | | | | |  |  |
| ***2020*** |  | ***Pre-Pandemic*** | ***First wave*** | ***Summer*** | ***Second wave*** |  |
| Cremation Records | F | 4.6 | 20.7 | 9.0 | 13.1 | 11.7 |
|  | M | 5.7 | 21.2 | 13.2 | 15.3 | 13.7 |
| Vital Statistics Records^2^ | F | -0.3 | 16.9 |  |  |  |
|  | M | 1.2 | 17.0 |  |  |  |
| ***2021*** |  | ***Third Wave*** |  |  |  |  |
| Cremation Records | F | 9.6 |  |  |  |  |
|  | M | 15.1 |  |  |  |  |

1 Absolute change refers to the difference between 2020 and the baseline (the average of 2017-2019).

2 The number of deaths in Ontario as reported by Statistics Canada in May 2021; these numbers are considered complete up to August 2020. ^3^

3 Number of cremations/deaths during 2020 compared to the number 2017-2019 divided by the average rate 2017-2019 multiplied by 100%.

1. **Age Analysis Tables**

**Table 2A**. A demonstration of the stability in the percent of Ontarians cremated by age, 2017-2020 and an analysis of the excess mortality detected in 2020-2021.

|  |  | **Timeframe (monthly)** | | | |  |
| --- | --- | --- | --- | --- | --- | --- |
|  | **Age group** | **January to March** | **April to**  **June** | **July to September** | **October to December** | **January – December** |
| **Number of Deaths** | |  |  |  |  |  |
| ***Baseline (2017-2019)^1^*** | |  |  |  |  |  |
| Cremation Records | 0 to 44 years | 713 | 748 | 804 | 793 | 3058 |
|  | 45 to 64 years | 3131 | 2982 | 3043 | 3078 | 12,234 |
|  | 65 to 84 years | 8528 | 7819 | 7600 | 8368 | 32,316 |
|  | 85 years & over | 6673 | 5596 | 5437 | 6329 | 24,036 |
| Vital Statistics Records^2^ | 0 to 44 years | 1188 | 1237 | 1305 | 1280 | 5,010 |
|  | 45 to 64 years | 4065 | 3858 | 3890 | 3942 | 15,755 |
|  | 65 to 84 years | 11,937 | 10,813 | 10,550 | 11,582 | 44,882 |
|  | 85 years & over | 11,350 | 9535 | 9202 | 10,602 | 40,688 |
| ***2020*** |  | ***Pre-Pandemic*** | ***First wave*** | ***Summer*** | ***Second wave*** |  |
| Cremation Records | 0 to 44 years | 852 | 987 | 1045 | 1027 | 3911 |
|  | 45 to 64 years | 3272 | 3450 | 3240 | 3407 | 13,369 |
|  | 65 to 84 years | 9102 | 9265 | 8500 | 9534 | 36.401 |
|  | 85 years & over | 6806 | 7035 | 5991 | 7241 | 27,073 |
| Vital Statistics Records^2^ | 0 to 44 years | 1290 | 1490 |  |  |  |
|  | 45 to 64 years | 4080 | 4320 |  |  |  |
|  | 65 to 84 years | 12,175 | 12,460 |  |  |  |
|  | 85 years & over | 11,125 | 11,475 |  |  |  |
| ***2021*** |  | ***Third Wave*** |  |  |  |  |
| Cremation Records | 0 to 44 years | 1028 |  |  |  |  |
|  | 45 to 64 years | 3455 |  |  |  |  |
|  | 65 to 84 years | 9780 |  |  |  |  |
|  | 85 years & over | 7155 |  |  |  |  |
| **Percent Cremated (%)** | | |  |  |  |  |
| 2017-2019^1^ | 0 to 44 years | 60.0 | 60.5 | 60.0 | 60.0 | 61.1 |
|  | 45 to 64 years | 77.0 | 77.3 | 77.0 | 77.0 | 77.6 |
|  | 65 to 84 years | 71.4 | 72.3 | 71.4 | 71.4 | 72.7 |
|  | 85 years & over | 58.8 | 58.7 | 58.8 | 58.8 | 59.0 |
| 2020 | 0 to 44 years | 66.0 | 66.2 |  |  | 69.0 |
|  | 45 to 64 years | 80.2 | 79.9 |  |  | 81.8 |
|  | 65 to 84 years | 74.8 | 74.4 |  |  | 76.5 |
|  | 85 years & over | 61.2 | 61.3 |  |  | 62.9 |

1 The average of the number and percent of deaths in 2017, 2018, and 2019 during the same time period.

**Table 2B**. A demonstration of the stability in the percent of Ontarians cremated by age, 2017-2020 and an analysis of the excess mortality detected in 2020-2021.

|  | **Age group** | **January to March** | **April to**  **June** | **July to September** | **October to December** | **January – December** |
| --- | --- | --- | --- | --- | --- | --- |
| **Absolute Change^3^**  ***2020*** | | | |  |  |  |
|  |  |  |  |  |  |  |
| Cremation Records | 0 to 44 years | 139 | 239 | 241 | 234 | 853 |
|  | 45 to 64 years | 141 | 468 | 197 | 329 | 1135 |
|  | 65 to 84 years | 574 | 1446 | 900 | 1166 | 4085 |
|  | 85 years & over | 133 | 1439 | 554 | 912 | 3037 |
| Vital Statistics Records | 0 to 44 years | 102 | 253 |  |  |  |
|  | 45 to 64 years | 15 | 462 |  |  |  |
|  | 65 to 84 years | 238 | 1647 |  |  |  |
|  | 85 years & over | -225 | 1940 |  |  |  |
| ***2021*** |  |  |  |  |  |  |
| Cremation Records | 0 to 44 years | 315 |  |  |  |  |
|  | 45 to 64 years | 324 |  |  |  |  |
|  | 65 to 84 years | 1252 |  |  |  |  |
|  | 85 years & over | 482 |  |  |  |  |
| **Percentage Increase^4^ (%)** | | | | | |  |
| ***2020*** |  |  |  |  |  |  |
| Cremation Records | 0 to 44 years | 19.5 | 31.9 | 30.0 | 29.5 | 27.9 |
|  | 45 to 64 years | 4.5 | 15.7 | 6.5 | 10.7 | 9.3 |
|  | 65 to 84 years | 6.7 | 18.5 | 11.8 | 13.9 | 12.6 |
|  | 85 years & over | 2.0 | 25.7 | 10.2 | 14.4 | 12.6 |
| Vital Statistics Records | 0 to 44 years | 8.6 | 20.5 |  |  |  |
|  | 45 to 64 years | 0.4 | 12.0 |  |  |  |
|  | 65 to 84 years | 2.0 | 15.2 |  |  |  |
|  | 85 years & over | -2.0 | 20.3 |  |  |  |
| ***2021*** |  |  |  |  |  |  |
| Cremation Records | 0 to 44 years | 44.2 |  |  |  |  |
|  | 45 to 64 years | 10.3 |  |  |  |  |
|  | 65 to 84 years | 14.7 |  |  |  |  |
|  | 85 years & over | 7.2 |  |  |  |  |

1 The average of the number and percent of deaths in 2017, 2018, and 2019 during the same time period.

2 The number of deaths in Ontario as reported by Statistics Canada in May 2021; these numbers are considered complete up to August 2020. ^3^

3 Absolute change refers to the difference between 2020/2021 records and the baseline (2017-2019).

4 Number of cremations/deaths during 2020 compared to the number 2017-2019 divided by the average rate 2017-2019 multiplied by 100%.
