# Supplementary material for: The Use of Cremation Data for Timely Mortality Surveillance During the COVID-19 Pandemic in Ontario, Canada: Validation Study
Source: JMIR Public Health Surveill. 2022 Feb 21;8(2):e32426. doi: 10.2196/32426 (PMC8862761; doi:10.2196/32426)
Supplement: Multimedia Appendix 3 [file publichealth_v8i2e32426_app3.docx]

**Multimedia Appendix 3.** Calculations for non–COVID-19 excess.

Following the identification of excess, the next question was whether COVID-19 deaths could account for the entirety of the excess. Cremation data contains a cause of death variable, permitting such analysis. This variable is an open-text, allowing the coroner to type in the cause of death. Using Python, version 3.8.0, the records were searched for the presence of COVID (or related terms) in the *cause of death*, *antecedent cause of death*, and *other cause of death* variables for deaths due to COVID-19. Deaths due to COVID-19 were then removed and excess was calculated and the population standardized increase from the baseline (2017-2019).

**Table S1**. Magnitude of non-COVID-19 excess mortality within the cremation record during the COVID-19 pandemic, January 2020 to March 2021.

| **Timeframe (monthly)** | **January to March** | **April to**  **June** | **July to September** | **October to December** | **January to December** |
| --- | --- | --- | --- | --- | --- |
| **Baseline (2017-2019)**^1^ |  |  |  |  |  |
| Number of cremations | 19,045 | 17,146 | 16,884 | 18,568 | 71,644 |
| **2020**  *(excluding COVID-19 deaths)* | ***Pre-Pandemic*** | ***First wave*** | ***Summer*** | ***Second wave*** |  |
| Number of cremations | 19,890 | 19,128 | 18,697 | 20,136 | 77,941 |
| Absolute Change^3^ | 935 | 1982 | 1813 | 1568 | 6297 |
| Population standardized percent increase (%)^4^  (95% CI)^5^ | 1.0  (-1.1 – 3.0) | 7.9  (5.7 – 10.1) | 7.5  (5.3 – 9.8) | 5.9  (3.9 – 8.1) | 5.6  (4.6 – 6.7) |
| **2021**  *(excluding COVID-19 deaths)* | ***Third wave*** | ***Third wave*** |  |  |  |
| Number of cremations | 18,215 | - | - | - | - |
| Absolute Change^3^ | -830 | - | - | - | - |
| Population standardized percent increase (%)^4^  (95% CI)^5^ | -7.6  (-9.4 – -5.7) | - | - | - | - |

1 The average of the number and percent of deaths in 2017, 2018, and 2019 during the same time period.

2 Cremation rates, analogous to mortality rates, were calculated as the number of cremations divided by the provincial quarterly population estimates published by Statistics Canada.^15^

3 Absolute change refers to the difference between 2020 and the baseline (2017-2019).

4 The population standardized percent increase is calculated as risk ratio (RR) – 1, where RR is the incidence of death (measured as the number of cremation) in the quarterly population estimates. The Q3 population estimate was used for the January-December risk ratio.

5 The 95% confidence interval for the percent increase is calculated as the (RR lower bound – 1)*100% to the (RRupper bound + 1)*100%. The RR confidence interval is calculated as =EXP(LN(RR)-(1.96*SE)), with SE(lm(rr)) = sqrt(1/Ncrem_(2017-19)_ - 1/Npop_(2017-19)_ +1/Ncrem_(2020)_ - 1/Npop_(2020)_))
